# Iron is a signal for *Stenotrophomonas maltophilia* biofilm formation, oxidative stress response, OMPs expression, and virulence

**DOI:** 10.3389/fmicb.2015.00926

**Published:** 2015-09-04

**Authors:** Carlos A. García, Eliana S. Alcaraz, Mirta A. Franco, Beatriz N. Passerini de Rossi

**Affiliations:** Cátedra de Microbiología, Facultad de Farmacia y Bioquímica, Universidad de Buenos AiresBuenos Aires, Argentina

**Keywords:** *Stenotrophomonas maltophilia*, iron, Fur, biofilms, oxidative stress response, IROMPs, virulence, DSF

## Abstract

*Stenotrophomonas maltophilia* is an emerging nosocomial pathogen. In many bacteria iron availability regulates, through the Fur system, not only iron homeostasis but also virulence. The aim of this work was to assess the role of iron on *S. maltophilia* biofilm formation, EPS production, oxidative stress response, OMPs regulation, quorum sensing (QS), and virulence. Studies were done on K279a and its isogenic *fur* mutant F60 cultured in the presence or absence of dipyridyl. This is the first report of spontaneous *fur* mutants obtained in *S. maltophilia*. F60 produced higher amounts of biofilms than K279a and CLSM analysis demonstrated improved adherence and biofilm organization. Under iron restricted conditions, K279a produced biofilms with more biomass and enhanced thickness. In addition, F60 produced higher amounts of EPS than K279a but with a similar composition, as revealed by ATR-FTIR spectroscopy. With respect to the oxidative stress response, MnSOD was the only SOD isoenzyme detected in K279a. F60 presented higher SOD activity than the wt strain in planktonic and biofilm cultures, and iron deprivation increased K279a SOD activity. Under iron starvation, SDS-PAGE profile from K279a presented two iron-repressed proteins. Mass spectrometry analysis revealed homology with FepA and another putative TonB-dependent siderophore receptor of K279a. *In silico* analysis allowed the detection of potential Fur boxes in the respective coding genes. K279a encodes the QS diffusible signal factor (DSF). Under iron restriction K279a produced higher amounts of DSF than under iron rich condition. Finally, F60 was more virulent than K279a in the *Galleria mellonella* killing assay. These results put in evidence that iron levels regulate, likely through the Fur system, *S. maltophilia* biofilm formation, oxidative stress response, OMPs expression, DSF production and virulence.

## Introduction

*Stenotrophomonas maltophilia* is a widespread environmental, multidrug resistant bacterium. It has become a nosocomial pathogen of increasing importance; in fact, it is the third most common nosocomial non-fermenting Gram-negative bacterium. Infection occurs principally in immunocompromised subjects, and in patients exposed to invasive devices and/or broad spectrum antibiotics (Looney et al., 2009; Brooke, 2012). *S. maltophilia* has also emerged as one of the most common isolated bacteria from the airway of cystic fibrosis (CF) patients (Pompilio et al., 2011; Vidigal et al., 2014).

Despite the broad spectrum of clinical syndromes associated with *S. maltophilia* infections, little is known about its virulence factors (Adamek et al., 2011). Factors that could be involved in the virulence of *S. maltophilia* include Smf1-fimbrial operon (de Oliveira-Garcia et al., 2003), protease StmPr1 (Windhorst et al., 2002; Nicoletti et al., 2011), exopolysaccharides and lipopolysaccharides (Huang et al., 2006), and siderophores (Garcia et al., 2012). Another important virulence factor of *S. maltophilia* is its capacity to form biofilms, communities of microbial cells that grow on biotic or abiotic surfaces embedded within extracellular polymeric substances (EPS) (Huang et al., 2006; Passerini de Rossi et al., 2007; Pompilio et al., 2008). *S. maltophilia* biofilms exhibit phenotypic characteristics that are distinct from those of planktonic organisms, including increased resistance to antimicrobial compounds (Di Bonaventura et al., 2004; Passerini de Rossi et al., 2009, 2012; Pompilio et al., 2010).

The genome of *S. maltophilia* K279a encodes a diffusible signal factor (DSF) dependent quorum sensing (QS) system that was first identified in *Xanthomonas campestris pv. campestris* (Xcc) (Fouhy et al., 2007; Huang and Wong, 2007). DSF synthesis is completely dependent on *rpfF*, which is part of the *rpf* operon (for regulation of pathogenicity factors) (Barber et al., 1997). Fouhy et al. (2007) demonstrated that the disruption of DSF signaling has pleiotropic effects in *S. maltophilia* K279a. The *rpfF* mutant had severely reduced motility, reduced levels of extracellular protease and altered LPS profiles. Their results showed that DSF controls aggregative and biofilm behavior and virulence in a nematode model. A recent study demonstrated that the significance of the *rpf* /DSF QS system is not confined to the virulence caused by *S. maltophilia* but also used by the plant-associated biocontrol agent *S. maltophilia* R551-3 (Alavi et al., 2013). Furthermore, the *S. maltophilia* QS signal is involved in interspecies signaling between different bacterial species within the CF lung and also has cross-kingdom antagonistic activity on *Candida albicans* (Ryan et al., 2008; Passerini De Rossi et al., 2014).

Besides QS signals, iron availability is a regulatory signal not only for the acquisition and utilization of this metal but also for the production of virulence factors in many pathogenic bacteria (Carpenter et al., 2009). Bacteria find iron limiting conditions in mammalian hosts, where free iron is limited and it is normally bound to sequestering proteins such as transferrin and lactoferrin. Thus, siderophores are considered important virulence factors for many pathogens allowing the microorganism to survive in the host. However, an excess of iron is toxic because of its ability to catalyze Fenton reactions and the formation of reactive oxygen species (ROS). In consequence, iron uptake has to be carefully regulated to maintain the intracellular concentration of the metal between desirable limits (Escolar et al., 1999). Iron-dependent gene regulation is mediated, in many bacterial species by Fur (ferric uptake regulator). Fur regulates the expression of iron uptake genes and is also involved in virulence and protection against oxidative stress (Carpenter et al., 2009). Fur is a global regulator that can act as either a repressor or an activator. Iron limitation induces or inhibits biofilm formation depending on the species (Wu and Outten, 2009).

Previous studies reported a relationship between Fur and the QS system. Fur positively regulates acyl homoserine lactone (AHL) production by *Pseudomonas syringae* pv. tabaci 11528 (Cha et al., 2008). In *Vibrio vulnificus* the gene *vvpE*, encoding the virulence factor elastase, is repressed under iron-rich conditions, and the repression was due to a Fur-dependent repression of *smcR*, a gene encoding a QS master regulator with similarity to *luxR* in *Vibrio harveyi* (Kim et al., 2013). Recently, evidence that iron limitation enhances AHL production was reported in *A. baumannii* (Modarresi et al., 2015).

*S. maltophilia* is an aerobic bacterium which generates ROS during metabolism. Superoxide dismutase catalyzes the dismutation of toxic superoxide radicals into molecular oxygen and hydrogen peroxide. Three SOD isoenzymes have been discovered, all prokaryotic organisms contain Mn-SOD or Fe-SOD while Cu/Zn-SOD is absent except for a few cases (Fridovich, 1978). Currently very little is known about the oxidative stress response of *S. maltophilia*.

The aim of this work was to assess the role of iron on *S. maltophilia* biofilm formation, EPS production, oxidative stress response, OMPs regulation, QS and virulence.

## Materials and methods

### Bacterial strains and culture conditions

The reference strain *Stenotrophomonas maltophilia* K279a, which genome is fully sequenced (GenBank: AM743169.1) was used in this study (Crossman et al., 2008). The spontaneous *fur* mutant, F60, derivative from the wild-type (wt) strain *S. maltophilia* K279a, was isolated in this study. *Xanthomonas campestris* pv. *campestris* (Xcc) 8004 and Xcc 8523 (*rpfF* mutant) were used in the DSF bioassay described by Barber et al. (1997). Strains were kept frozen at −20°C in 15% glycerol. Before use, bacteria were cultured on tryptone soya agar (TSA; Oxoid Ltd, Basingstoke, Hampshire, UK) for 24 h at 35°C. Unless otherwise stated, all cultures were grown in tryptone soya broth (TSB, Oxoid Ltd) in the presence or absence of 200 μM 2,2′-dipyridyl (Dip; Sigma-Aldrich), and incubated for 48 h at 35°C. When required, the cultures were vigorously aerated on a gyratory water bath shaker (Model G75, New Brunswick Scientific Co. Edison NJ, USA) at 200 r.p.m.

### Isolation of *fur* mutants

The manganese mutagenesis technique (Hantke, 1987) with few modifications was used to isolate spontaneous *fur* mutants of *S. maltophilia*. Briefly, an overnight culture of K279a in LB broth was plated on LB agar containing 20 mM MnSO_4_ and 200 μM Dip. After 72 h of incubation, the robustly growing manganese-resistant colonies were tested on chrome azurol S (CAS) agar plates prepared with the modifications described previously by our group (Garcia et al., 2012) and supplemented with 20 mM FeCl_3_, to identify *fur* mutants by constitutive siderophore production. In order to confirm the mutations, the full-length *fur* gene and 100 bp upstream from the start codon were PCR amplified using primers designed in this study (For 5′-GGCGGTTGGGGAAATCAAAC-3′ and Rev 5′-CAACGAAAAACCCCGGGCA-3′) and sequenced. PCR was performed using *Taq* DNA polymerase (Promega Corporation) and chromosomal DNA from *S. maltophilia* putative *fur* mutants as template under the following conditions: 1 cycle of 96°C for 5 min; 30 cycles of 96°C for 1 min, 48°C for 30 s, and 72°C for 1 min, and a final cycle of 72°C for 7 min (Biometra, TPersonal48). DNA sequences were determined at Unidad de Genómica (Instituto de Biotecnología, Instituto Nacional de Tecnología Agropecuaria-INTA, Argentina). The nucleotide sequences were edited using the BioEdit Sequence Alignment Editor (Hall, 1999) and sequences were aligned with MEGA v4.0 (Tamura et al., 2007).

### Biofilm formation assay

Biofilms were prepared using a static microtitre plate model as previously described (Passerini de Rossi et al., 2009). Briefly, overnight cultures of *S*. *maltophilia* strains were standardized to contain approximately 10^6^ CFU/ml. For each test condition (presence or absence of 200 μM Dip in TSB medium), 8 wells of a sterile flat-bottom 96-well polystyrene microtiter plate (TPP, Trasandingen, Switzerland) were filled with 200 μl of the standardized inoculum. Uninoculated medium controls were included. After 48 h incubation, the final culture density was determined by measuring the OD_546_ using a Multiskan EX plate reader (Thermo electron corporation, Hudson, United States). Then, the culture medium was removed from each well and plates were washed three times with phosphate buffered saline (PBS) to remove non-adherent cells. Biofilms were stained with 0.01% crystal violet (CV; Mallinckrodt, Chemical Works, NY, USA) for 30 min. The plates were washed, and the dye bound to the biofilm was extracted with ethanol 95%. The total biomass (attached cells and extracellular matrix) was quantified by measuring the OD_546_ of dissolved CV. Results were expressed as the level of CV staining relative to the final culture density [CV (OD_546_)/Growth (OD_546_)], to avoid variations due to differences in bacterial growth generated by the chelator.

### Confocal laser scanning microscopy (CLSM)

For confocal microscopy biofilms were formed on Nunc Lab-Tek 8-well chamber slide (No. 155411) containing a borosilicate glass base 100 μm thick. Overnight cultures of K279a and F60 were standardized to contain approximately 10^6^ CFU/ml and for each test condition (presence or absence of 200 μM Dip), chambers were filled with 400 μl of the standardized inoculum. After 48 h of incubation, the wells were rinsed with sterile physiological saline (0.9% NaCl) in order to eliminate any non-adherent bacteria. The wells were refilled with physiological saline containing 2.5 μM Syto9® (Molecular Probes, Grand Island, NY), a green fluorescent nucleic acid marker, and incubated in the dark for 20 min. Images were acquired with a confocal laser-scanning microscope Carl Zeiss LSM510-Axiovert 100 M by sequentially scanning with a 488 nm argon laser using a 40X water immersion objective lens, at the Instituto de Investigaciones Biomédicas (Pontificia Universidad Católica Argentina-CONICET, Argentina). Emitted fluorescence was recorded within the 505–530 nm range to visualize Syto9 fluorescence, and Z-stacks were captured every10 μm at different areas in the well. Images were analyzed using the ZEN 2009 Light Edition (Carl Zeiss) and three-dimensional projections of the biofilms' structure were reconstructed using the ImageJ program (ImageJ. Available online: http://rsbweb.nih.gov/ij/).

### Extracellular polymeric substances (EPS) production assays

The EPS production was measured by ethanol precipitation as previously described (Boon et al., 2008) with some modifications. *S. maltophilia* strains were cultured in 100 ml of LB supplemented with 0.1% glucose (LB-glc) in the presence or absence of 200 μM Dip. After 48 h of incubation with shaking, the supernatants were collected by centrifugation at 14,000 r.p.m for 20 min. The pellets were dried overnight at 56°C before determination of dry weight of the biomass. After filter sterilization, supernatants were mixed with 2 volumes of absolute ethanol and incubated at −20°C overnight. The precipitated EPS were centrifuged and dried overnight at 56°C. Results were expressed as the amount of EPS (μg) relative to the dry weight of the biomass [EPS (μg)/Biomass (mg)]. The carbohydrate content in EPS was quantified by the phenol-sulfuric acid method (Dubois et al., 1956) with glucose as the standard, and the protein content was measured by the Bradford method (Bradford, 1976) using bovine serum albumin (Sigma-Aldrich) as standard.

### Attenuated total reflection fourier transform infrared (ATR-FTIR) spectroscopy

ATR-FTIR spectroscopy was performed with *S. maltophilia* ethanol-precipitated EPS samples. Infrared spectra were recorded using a Nicolet 380 FT-IR (Thermo Scientific Electron Corporation) with an ATR accessory equipped with a 45° single-reflection ZnSe prism. Thin particle films were prepared by placing a suspension of dried EPS in Milli-Q water (7.5 mg of EPS in 150 μl of water) on the ZnSe prism and drying for 60 min. Absorption spectra were recorded between 4000 and 900 cm^−1^ with a resolution of 4 cm^−1^ and co-addition of 32 scans. Recording of spectra, data storage and data processing were performed using the OMNIC software version 7.2 (Thermo Scientific Electron Corporation).

### Microscopy analysis of matrix exopolysaccharides

The presence of exopolysaccharides in the matrix of *S. maltophilia* biofilms was detected as previously described (Passerini de Rossi et al., 2009). Sterile microscope borosilicate coverslips were aseptically placed into Petri plates along with 15 ml TSB inoculated with *S. maltophilia* strains (10^6^ CFU/ml) and incubated for 48 h. The coverslips were removed, rinsed with distilled water and stained in the dark with 0.1% calcofluor white dye (Fluorescent brightener 28, Sigma-Aldrich) for 10 min. Then, coverslips were rinsed, mounted on the microscope slides and examined with an Olympus BX50-DP73 microscope (Olympus, New York, USA). Images were obtained with a 40X lens objective. Calcofluor emissions were detected using a DAPI filter (excitation/emission wavelengths: 330–385/420 nm). The polysaccharide matrix fluoresces blue under the DAPI light filter.

### Determination of SOD isoenzymes

The determination of the SOD isoenzymes in *S.maltophilia* was performed using inhibition methods according to Dunlap and Steinman (1986). First, crude extracts were prepared from planktonic cultures. *S*. *maltophilia* cells from TSB or TSB-Dip 48 h-cultures were harvested by centrifugation, washed and suspended in 7 ml of PBS with 0.25 mM PMSF (Fluka Biochemika). Suspensions were sonicated using two 3 min pulses (Vibra cell, Sonics & Materials Inc. Danbury, Connecticut, USA) and centrifuged at 10,000 r.p.m. for 10 min at 4°C. The supernatant protein concentration was determined by the Bradford assay. Proteins were separated by using a 10% non-denaturing PAGE in the presence of 50 mM Tris, 300 mM glycine, and 1.8 mM EDTA at 120 V. Gel lanes were loaded with 20 μg total protein and purified MnSOD from *Escherichia coli* (Sigma-Aldrich) was used as standard. The metal present in the active site of SOD molecule was determined using inhibition methods. Gels were treated with 10 mM NaCN or 3.7 mM H_2_O_2_ to inactivate Cu/ZnSOD or FeSOD, respectively. Resistance to both cyanide and H_2_O_2_ is characteristic of MnSODs. SOD activity was visualized by staining with NBT as described by Beauchamp and Fridovich (1971). Briefly, after inhibition assays gels were incubated with shaking for 30 min in the dark in a solution of 500 μM NBT (Sigma Aldrich), 50 mM potassium phosphate buffer pH 7.8, 1 mM EDTA, 20 mM TEMED (Invitrogen, Carlsbad, CA, USA) and 30 μM riboflavin (Sigma-Aldrich). The gels were illuminated until achromatic zones indicating SOD activity were visible in a uniformly blue background.

### SOD activity assay

The SOD activity was determined by the riboflavin/methionine system (Beauchamp and Fridovich, 1971) in crude extracts from planktonic (see Determination of the SOD isoenzymes**)** and biofilm cultures of *S. maltophilia* strains under iron replete (TSB) or iron restricted conditions (TSB-Dip). Biofilm crude extracts were obtained by using 12-well microtiter plates. Wells were filled with 3 ml of the standardized inoculum (ca. 10^6^ CFU/ml). After 48 h of incubation, wells were aspirated, washed and then filled with 1 ml of PBS. Adherent cells were detached using a sterile cell scraper and the resulting cell suspension from 9 wells was centrifuged. The pellet was suspended in 1 ml of PBS with 0.25 mM PMSF. Then, crude extracts were obtained and their protein concentration was determined as described. For SOD activity determination, aliquots of 100 μl from each extract were treated with 300 μl of 13 mM methionine (Sigma-Aldrich), 100 μl of 1 mg/ml NBT, 300 μl of 100 nM EDTA, and 300 μl of 0.5 mM riboflavin in the presence of light. After 15 min, OD_560_ was determined. A unit of SOD was defined as the quantity of enzyme required to produce a 50% inhibition of NBT reduction. Activity was expressed as units of SOD per mg of protein [SOD (U)/Total protein (mg)].

### Nitro blue tetrazolium (NBT) assay

The NBT assay was performed as described by Aiassa et al. (2010) with the following modifications. *S. maltophilia* biofilms formed in 96-well microtiter plates under iron replete or iron restricted conditions, as described above, were washed two times with PBS, then 100 μl NBT (1 mg/ml) were added to each well. After incubation for 30 min at 37°C in the dark the reaction was stopped with 20 μl of 0.1 N HCl. Each well was treated with 50 μl of DMSO (Calbiochem, La Jolla, CA, USA) to extract the reduced NBT. Reduced NBT was measured as formazan blue at 540 nm. Results were expressed as reduced NBT relative to the biomass of biofilm normalized to cell density [NBT (OD_546_)/Biomass (CV/OD)].

### SDS-PAGE of OMP-enriched fractions

*S*. *maltophilia* cells from 48 h-cultures in TSB or TSB-Dip were harvested by centrifugation (15 min, 4°C, 10,000 r.p.m) and used for preparation of Sarkosyl-insoluble OMP-enriched fractions as described previously (Passerini De Rossi et al., 2003). SDS gel electrophoresis was carried out according to the Tris/tricine method (Schagger and Von Jagow, 1987). Gradient gels of 4–20% (1 mm thick) were used. Gels were stained with Coomassie brilliant blue R-250 (Sigma-Aldrich). A high molecular weight standard mixture for SDS gel electrophoresis (SDS-6H, Sigma-Aldrich) was used as molecular size standards.

### Mass spectrometric analysis of proteins

Selected bands were cut from the SDS-polyacrylamide gel stained with Coomassie blue and the proteins were subjected to enzymatic hydrolysis with 25 ng/μl trypsin. The samples were analyzed at the Laboratorio Nacional de Investigación y Servicios en Péptidos y Proteínas (LANAIS–PRO, CONICET-UBA). The sizes of the peptide fragments generated were determined by mass spectrometry on a LCQ Duo ESI/TRAP (Thermo Fisher) after separation by HPLC with a column Vydac RP-C18 (1.0 × 200 mm). The data were used to interrogate the National Center for Biotechnology Information nonredundant protein data bases by using the MASCOT MSMS program (Matrix Science) available at www.matrixscience.com.

### DSF production

The bioassay described by Barber et al. (1997) was generally followed. DSF production was assayed by measuring the restoration of endoglucanase activity to the *rpfF* mutant Xcc 8523 by extracts from culture supernatants. To obtain the extracts, *S. maltophilia* strains were grown in Stainer-Scholte (SS) minimal medium plus 0.1% casamino acids (Difco laboratories, Detroit, MI, USA) (SSC, iron-limited condition) and in SSC supplemented with 100 μM FeCl_3_ (iron rich condition). After 48 h of incubation the final culture density was adjusted to an OD_540_ of 1.00 to avoid variations due to differences in bacterial growth generated by iron supplementation. Then, the cells were removed by centrifugation, the supernatants were acidified to pH 3.0 with chlorhydric acid and loaded onto 30 mg Oasis MAX columns (Waters Oasis® Milford, USA). The column was washed with 5 mM sodium acetate:methanol (95:5) and elution was performed with methanol followed by 2% acetic acid in methanol. The extracts were evaporated and suspended in 500 μl sterile water for the quantitative assay for the DSF. Diameters of zones of carboxymethyl cellulose (CMC; Sigma-Aldrich) hydrolysis, produced by 50 μl of extract, were measured and converted to relative endoglucanase units with a standard curve constructed using dilutions of a standard of cellulase I (Sigma Aldrich). One unit of endoglucanase was defined as the amount which gave a hydrolysis zone of 12 mm diameter.

### *Galleria mellonella* killing assay

The virulence of *S. maltophilia* strains was evaluated by infecting larvae of the wax moth *G. mellonella* as described by Seed and Dennis (2008) with some modifications. First, in order to establish the optimal inoculum of *S. maltophilia* required to kill *G. mellonella* over 24–96 h, caterpillars of 250–350 mg in weight with a cream-colored cuticle, were inoculated with 10 μl of K279a suspensions containing 10^4^, 10^5^, and 10^6^ CFU. Bacterial suspensions were obtained from overnight TSA cultures. The cells were washed twice with physiological saline and diluted to obtain the different inocula. The concentration of each inoculum was confirmed by colony counting on TSA plates. Bacteria were injected into the hemocoels of the caterpillars via a left proleg using 20 μl Hamilton syringes. Caterpillars were incubated in Petri dishes lined with filter paper at 30°C for 96 h and scored for survival daily. Insects were considered dead when they displayed increased melanization and failed to respond to touch. Second, *G. mellonella* killing assays were performed on K279a and F60 using the determined optimal inoculum. In all experiments, 12 caterpillars were used for each condition, including a control group of caterpillars inoculated with physiological saline to monitor for killing due to physical trauma. Experiments that had more than one dead caterpillar in the control group were discarded and repeated. Survival curves were plotted using the Kaplan-Meier method, and differences in survival were calculated by using the log-rank test (Graph Pad Prism version 5.0, Software Inc., La Jolla, CA).

### Statistical analysis

All experiments were performed at least in triplicate and repeated on three different occasions. Statistical analysis was performed using GraphPad InStat version 3.01 for Windows (GraphPad Software, San Diego California USA, www.graphpad.com). Results were analyzed by One-Way ANOVA with Dunnett's post-test, and differences were considered significant at *p* < 0.05.

## Results and discussion

In order to assess the role of iron on the production of biofilms and factors potentially involved in biofilm formation and virulence of *S. maltophilia* the following studies were performed under iron-limiting and iron-replete conditions. Iron-dependent gene regulation in bacteria is generally mediated by the Fur system (Escolar et al., 1999). To our knowledge, *S. maltophilia fur* mutants have not yet been obtained. Thus, we carried out the manganese-induced mutagenesis of the wt strain K279a to select *fur* mutants and test the possible role of Fur in iron dependent regulation of these factors.

### Isolation of *S. maltophilia fur* mutants

The genome of *S. maltophilia* K279a (GenBank: AM743169.1) revealed a gene SMLT_RS09600 (old locus tag Smlt1986, putative *fur* gene) encoding a 135 amino acid Fur family transcriptional regulator.

Sequence similarity searches of the available nucleotide databases were performed with the BLASTN program (http://www.ncbi.nlm.nih.gov/blast). The putative *fur* gene is highly conserved in *S. maltophilia*, SMLT_RS09600 from K279a is 99–96% identical to putative *fur* genes of *S. maltophilia* strain 13637 (Accession: CP008838, region: 2056865–2057272), *S. maltophilia* JV3 (Accession: CP002986, region: 1789094–1789500), and *S. maltophilia* R551-3 (Accession: CP001111, region: 1790286–1790693). Furthermore, K279a putative *fur* gene is 86% identical to Xcc ATCC 33913 *fur* (XCC1470) and is 76% identical to *Pseudomonas aeruginosa* PAO1 *fur* (PA4764).

These data prompted us to carry out the manganese selection technique (Hantke, 1987) to select spontaneous *fur* mutants of *S. maltophilia*. This method has been successfully used to isolate *fur* mutants in other Gram-negative bacteria (Hantke, 1987; Passerini De Rossi et al., 2003). A total of 51 independent clones from K279a were obtained on manganese LB agar. A clone, named F60, showed constitutive siderophore production. To discard reversion back to the wild type phenotype, the mutant was tested for the deregulated phenotype on CAS agar plates supplemented with iron several additional times with consistent results.

With the aim of confirming the presence of mutations in F60, a pair of primers were designed to PCR amplify the full-length *fur* gene and 100 bp upstream from the start codon. Sequenced PCR products revealed a point mutation (T
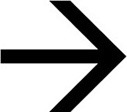
C) 65 bp upstream of the ATG initiation codon, in the promoter region located within the −10 sequence.

### Iron as a signal for biofilm formation

**Figure 1:**
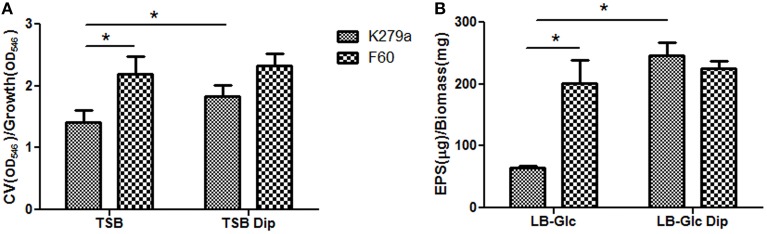
**Biofilm formation and production of EPS by *S. maltophilia* K279a and its Fur mutant. (A)** Biofilm formation assessed by microplate colorimetric assay. Biofilms were grown in TSB with or without 200 μM Dip for 48 h in static 96-well polystyrene plates. The biomass of biofilms was quantified by CV staining and expressed relative to the final culture density. **(B)** Production of EPS. *S. maltophilia* K279a and F60 were cultured in LB supplemented with 0.1% glucose (LB-glc) in the presence or absence of 200 μM Dip for 48 h at 35°C. Supernatants were mixed with 2 volumes of absolute ethanol and the precipitated EPS were dried overnight at 56°C before determination of dry weights. Results were expressed as the amount of EPS relative to the dry weight of the biomass. Results represent the mean ± standard deviation of one representative experiment. Asterisks indicate significant difference (^*^p < 0.01).

In order to assess the role of iron on *S. maltophilia* biofilm formation, K279a and its *fur* mutant were grown for 48 h in TSB in the presence or absence of Dip in polystyrene microtiter plates. The growth yield of F60 in these media was similar to that of K279a (data not shown). Biofilm formation was quantified by crystal violet (CV) staining. Figure 1A shows that K279a was significantly more efficient in producing biofilms in the presence of Dip than in TSB (*p* < 0.05). On the other hand, under iron-replete conditions the amount of biofilm produced by the *fur* mutant was higher than that of the wt strain (*p* < 0.05), and the addition of the iron chelator did not affect its biofilm production.

**Figure 2:**
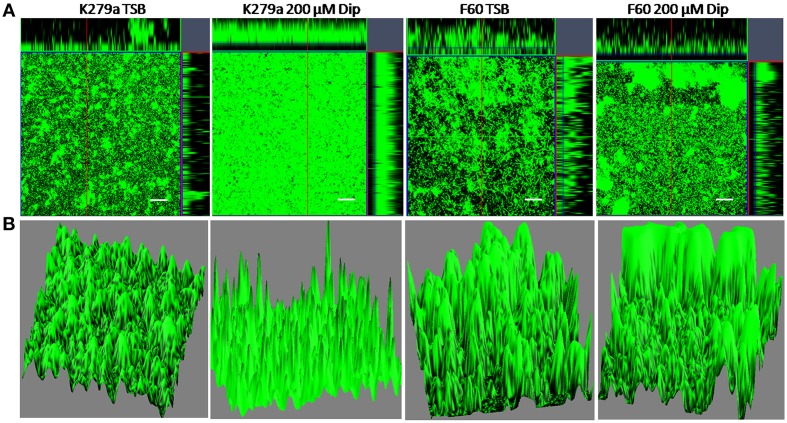
**CLSM analysis of biofilms from *S. maltophilia* K279a and its Fur mutant**. Biofilms were grown in glass LabTek chambers, in TSB and TSB supplemented with 200 μM Dip for 48 h. Then, biofilms were stained with Syto9 and analyzed by CSLM, using a 40X water immersion objective lens. **(A)** Central panels represent the x–y plane, and the top and right side panels represent the x–z and y–z planes, respectively. Scale bars represent 20 μm. **(B)** Three-dimensional projections of biofilm structure were reconstructed using the Image J program. The images are representative of those obtained on three independent occasions.

To further investigate the role of iron on biofilm formation, CLSM was used to analyze the architecture of the 48 h-biofilms stained with Syto9. Figure 2A shows confocal images acquired from K279a and F60 biofilms. K279a biofilm grown in TSB presented a confluent growth with microcolonies scattered on the surface (x–y plane) and the *z*-projection of the *x*–*y* stacks revealed variable thickness. In the presence of Dip, K279a produced a much more compact biofilm with enhanced thickness. On the other hand, F60 biofilms grown under both iron conditions showed larger microcolonies and were thicker than biofilms from K279a grown in TSB. Figure 2B shows three-dimensional reconstructions obtained from confocal stack images using the ImageJ program. The architecture of biofilms consisted of peaks interlaced with water channels. K279a biofilm presented peaks with a range of 20–30 μm in height while iron restriction resulted in the formation of taller peaks up to 50 μm. Irrespective of culture conditions F60 formed biofilms with more and taller peaks of up to 90 μm in height.

Since the growth yield of F60 was similar to that of K279a, the phenotypic differences observed between these strains were not due to a growth defect. Our results put in evidence that iron levels regulate, possible through the Fur system, *S. maltophilia* biofilm formation and architecture.

Iron limitation induces or inhibits biofilm formation depending on the species (Wu and Outten, 2009). The presented data show that in *S. maltophilia* iron restriction induces biofilm formation, a similar behavior to that previously reported for *Legionella pneumophila, Acinetobacter baumannii* and *Staphylococcus aureus* (Tomaras et al., 2003; Johnson et al., 2005; Hindre et al., 2008; Modarresi et al., 2015).

### Effect of iron on extracellular polymeric substances production and chemical composition

The production of extracellular polymeric substances (EPS) in planktonic cultures grown under iron-limiting and iron-replete conditions was quantified by ethanol precipitation. Results showed that F60 produces 3-fold higher amounts of EPS than K279a, and the presence of Dip improved the EPS production only in the wt strain (Figure 1B). Hence, iron limitation, likely through Fur, increased EPS production in *S. maltophilia*. This is in accordance with the enhanced alginate production detected in *P. aeruginosa* under iron limitation (Wiens et al., 2014).

**Figure 3:**
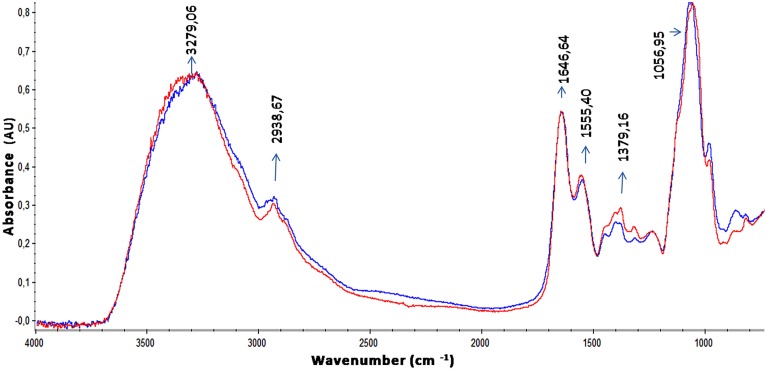
**ATR-FTIR spectra of EPS from *S. maltophilia* strains**. Spectra of ethanol precipitated EPS from K279a and F60 are shown in blue and in red, respectively. Bands associated with lipids (3100–2800 cm^−1^), proteins (1700–1500 cm^−1^) mainly identified by the amide I (1646,64 cm^−1^) and amide II bands (1555,40 cm^−1^), and polysaccharides and nucleic acids (1300–900 cm^−1^) are indicated in the spectra.

The EPS of the majority of bacterial biofilms including *P. aeruginosa* consists mainly of polysaccharides, proteins, and nucleic acids (Laverty et al., 2014). The chemical composition of the *S. maltophilia* EPS fractions was assessed by measuring the total carbohydrate and protein contents (Table 1). Interestingly, EPS from K279a and F60 had a similar carbohydrate/protein ratio (~0.50). To gain more insight, at the biochemical composition level, the EPS of both strains were also compared using ATR-FTIR spectroscopy. Figure 3 shows the bands associated with lipids (3100–2800 cm^−1^), proteins (1700–1500 cm^−1^), and polysaccharides and nucleic acids (1300–900 cm^−1^) (Jiao et al., 2010). The FTIR spectra revealed no significant variations in relative abundance of the components between the two EPS fractions. These results put in evidence that F60 produces a higher amount of EPS than K279a in the absence of Dip, but with a similar biochemical composition.

**Table 1:** **Macromolecular composition of EPS extracted from *S. maltophilia* strains**.

**Strains**	**EPS (μg EPS/mg Biomass)**	Chemical analysis of EPS^*^		
		**Carbohydrates (mg glc/mg EPS)**	**Proteins (mg proteins/mg EPS)**	**Carbohydrate/Protein (mg glc/mg protein)**
K279a	64.9 ± 3.3	0.25 ± 0.02	0.49 ± 0.03	0.51
F60	201.1 ± 38.0	0.26 ± 0.05	0.52 ± 0.05	0.50

*Results represent the mean ± standard deviation of one representative experiment*.

* *Carbohydrates and proteins were determined by the phenol-sulfuric acid method (Dubois et al., 1956) and the Bradford method (Bradford, 1976), respectively*.

**Figure 4:**
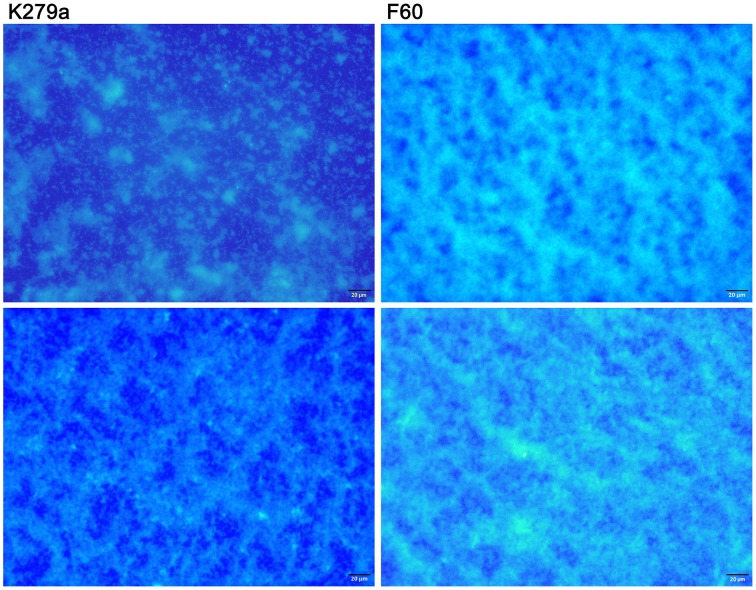
**Microscopy detection of matrix exopolysaccharides in *S. maltophilia* biofilms**. K279a and F60 biofilms formed on borosilicate coverslips in TSB (upper panel) or TSB-Dip (lower panel) for 48 h were stained with calcofluor white and examined by epifluorescence microscopy. The polysaccharide matrix fluoresces blue under the DAPI light filter. Calcofluor stains cells as discrete points while EPS is visible as a continuous sheet. Biofilms were viewed at 400X magnification.

The EPS matrix is important in forming the biofilm architecture and in protecting bacteria from antimicrobials and host defense mechanisms. Exopolysaccharides, fundamental components of EPS, are recognized as virulence factors (Cescutti et al., 2011; Laverty et al., 2014). In order to detect the presence of exopolysaccharides in the matrix of *S. maltophilia* biofilms samples stained with calcofluor white were examined with epifluorescence microscopy. Figure 4 shows micrographs from K279a and F60 biofilms formed in TSB and TSB-Dip. Samples stained with calcofluor white showed that the cells and microcolonies of both strains attached to borosilicate were embedded within a blue fluorescent material. According to the higher amounts of EPS produced by F60, the mutant formed more compact structures, resembling cotton wool, than K279a. Again, under iron-restricted conditions, the biofilm from K279a showed the presence of EPS as an almost continuous sheet.

We have previously reported that binding of calcofluor white indicates that β-linked polysaccharides, such as cellulose and chitin, are part of the matrix of *S. maltophilia* biofilms (Passerini de Rossi et al., 2007). This is in accordance with the presence of many β-1-4 unions in the primary structure of *S. maltophilia* exopolysaccharides characterized by Cescutti et al. (2011) from planktonic cultures of two mucoid clinical isolates obtained from two CF patients. This is a novel structure among bacterial polysaccharides: It has three uronic acid residues on a total of four sugars in the repeating unit and bears an additional negative charge due to the d-lactate substituent. The authors suggested that the abundance of negative charges, a common feature of the exopolysaccharides produced by two other bacteria infecting CF patients (*P. aeruginosa* and *Inquilinus limosus*), is a characteristic which somehow constitutes an advantage for the microbes in the lung environment.

Xiao et al. (2012) reported that exopolysaccharides modulate the development and spatial distribution of microcolonies in *Streptococcus mutans* biofilms. These authors suggested that individual microcolonies encased in polysaccharides may serve as architectural units that become connected during biofilm construction, forming compartmentalized networks that confer highly heterogeneous yet cohesive environments within the 3D architecture. CLSM analysis demonstrated that, under iron restriction, K279a produces a much more compact biofilm with enhanced thickness and 3D organization, similar to that of F60 biofilms. These results could be in part due to an increment in EPS production.

### Role of iron on the oxidative stress response of *S. maltophilia*

*S. maltophilia* is an aerobic bacterium which generates reactive oxygen species (ROS) during metabolism. Aerobic bacteria prevent the oxidative stress by producing antioxidant enzymes including superoxide dismutases (SODs). Superoxide dismutases is a family of three metalloenzymes containing manganese (MnSOD) or iron (FeSOD) or both copper and zinc (Cu/ZnSOD) cofactors. FeSOD and MnSOD are generally evidenced in prokaryotes (Fridovich, 1978).

First, we decided to determine the type of SOD isoenzymes produced by *S. maltophilia* K279a using inhibition methods (Dunlap and Steinman, 1986). Crude extracts of planktonic cells of *S*. *maltophilia* K279a cultured in TSB or TSB-Dip were separated by using a 10% non-denaturing PAGE. A single band was seen under both growth conditions and inhibition experiments showed that MnSOD is the only SOD isoenzyme present since this band was inhibited neither by H_2_O_2_ nor by NaCN (data not shown). The mobility of the K279a MnSOD differs from that of the MnSOD purified from *E. coli* used as a reference; this fact could reflect some structural differences between them. Analysis of the *S. maltophilia* K279a genome (GenBank: AM743169.1) showed the presence of five *sod* genes, two of them, SMLT_RS13450 and SMLT_RS15415 coding for putative MnSODs. This was the only isoenzyme detected under the experimental conditions used in this study. Our result is in accordance with the report of the expression of only MnSOD by the quinclorac-degrading strain *S. maltophilia* WZ2 (Lü et al., 2009). This fact allowed us to study the role of iron in SOD regulation by determining the total SOD activity by the riboflavin/methionine system (Beauchamp and Fridovich, 1971).

**Figure 5:**
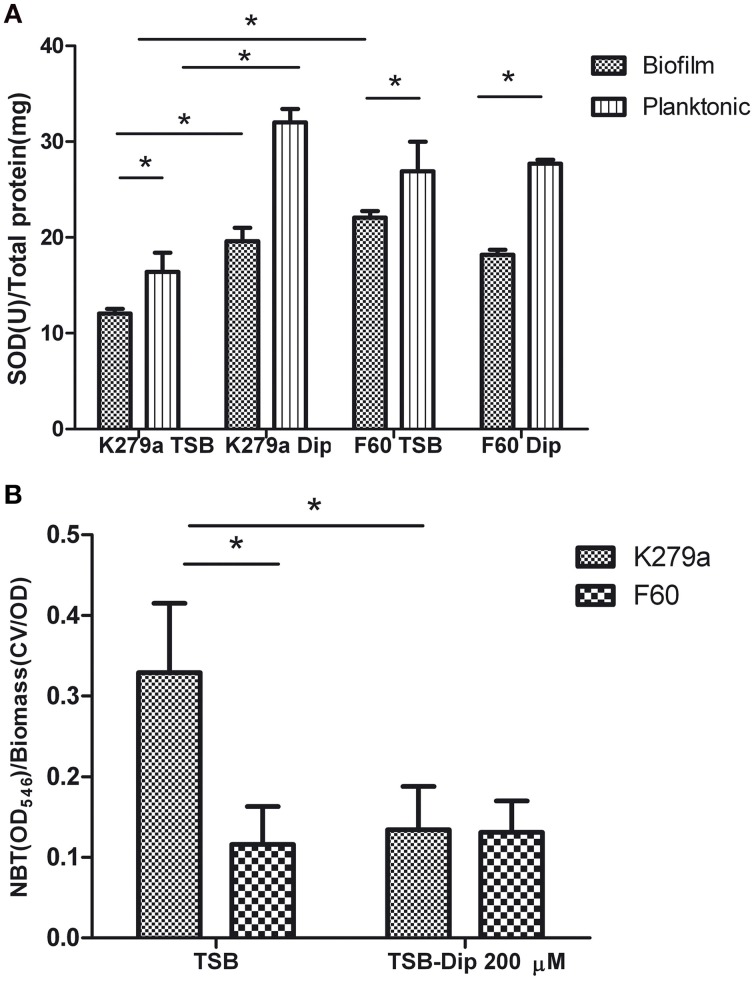
**SOD activity and ROS levels in *S. maltophilia* K279a and F60**. Bacterial cultures were performed in TSB and TSB supplemented with 200 μM Dip for 48 h **(A)** SOD activity of crude extracts from plaktonic and biofilms cells determined by the riboflavin/methionine system, as described in the text. Activity was expressed as units of SOD per mg of protein. **(B)** Levels of ROS in biofilms of *S. maltophilia*. The biofilms formed in microtiter plates were treated with NBT. Reduced NBT was measured as formazan blue at 540 nm and expressed relative to the biomass of biofilm. Results represent the mean ± standard deviation of one representative experiment. Asterisks indicate significant difference (^*^p < 0.01).

Comparative studies of SOD activity were conducted on crude extracts obtained from biofilms and planktonic cultures of K279a and F60 in the presence or absence of Dip (Figure 5A). The use of 12-well microtitre plates instead of 96-well plates for biofilm formation allowed the recovering of larger amounts of biomass needed for the assay. The crude extracts obtained from the biofilm of K279a in the absence of Dip showed the lowest total SOD activity (12.06 ± 0.5 U/mg of protein), and iron deprivation produced a significant increase in SOD activity (19.6 ± 1.4 U/mg of protein). On the other hand, the SOD activity in crude extracts of F60 biofilms obtained in the presence or absence of Dip (22.06 ± 0.7 and 18.20 ± 0.5 U/mg of protein, respectively) was higher than that of K279a cultured in the absence of Dip. The SOD activity from planktonic cultures of K279a and F60 under both conditions showed the same behavior. In conclusion, these results demonstrate that SOD activity in *S. maltophilia* is negatively regulated by iron, likely through Fur. In many organisms Fur regulates the expression of the *sodA* gene which encodes MnSOD. Our results are in agreement with those of Hassett et al. (1996) who reported that, the addition of Dip to the wt *P. aeruginosa* PAO1 produced an increase in MnSOD activity, similar to the *fur* mutant phenotype. Interestingly, the SOD activity of biofilms was lower than that of the respective planktonic counterparts, a fact that could be due to the lower metabolic activity of biofilms.

Finally, the production of ROS by *S. maltophilia* in biofilms grown under iron replete or iron restricted conditions was evaluated by the NBT assay (Aiassa et al., 2010). Figure 5B shows that the biofilm of K279a cultured in the presence of Dip had 2.5-fold lower levels of ROS than biofilms cultured in TSB. The biofilms of F60 formed under both conditions showed levels of ROS similar to that of K279a cultured in TSB-Dip. These results are in concordance with the respective levels of SOD activity in the biofilm (Figure 5A). Thus, the formation of biofilms under low iron conditions led to low ROS generation. A similar result was obtained when the production of ROS was determined in supernatants of planktonic cultures (data not shown). One limitation of these results is that the methodology used for evaluating ROS production is not quite specific. Further studies with specific fluorescent probes for detection of superoxide radicals or hydrogen peroxide, as well as electron spin resonance (ESR) spectrometry, are needed to quantify and identify these radicals.

### Identification of iron regulated OMPs by mass spectrometry

Under iron-limiting growth conditions many bacteria express high affinity systems to scavenge this metal from different sources. In Gram-negative bacteria specific outer membrane receptors bind FeIII-siderophore complexes, which are generally internalized into the periplasm across the outer membrane with energy transduced by the TonB system (TonB/ExbB/ExbD complex) from the cytoplasmic membrane (Braun, 1995). Furthermore, many of these genes are repressed by Fur (Crosa, 1997).

**Figure 6:**
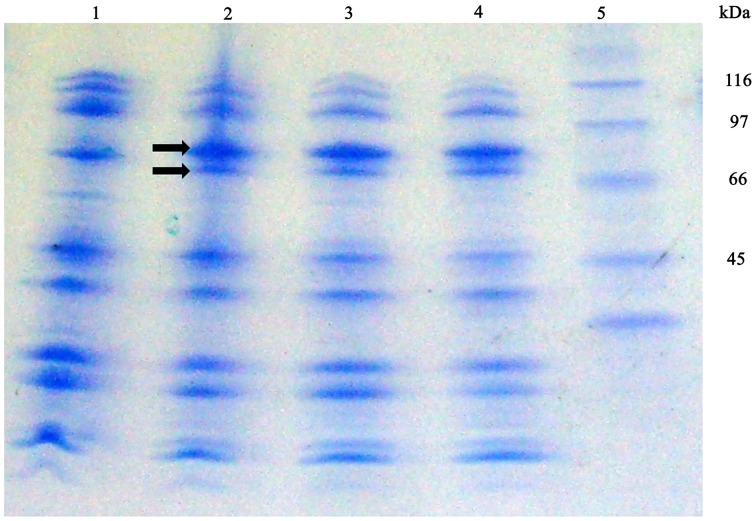
**SDS-PAGE profiles of Sarkosyl-insoluble OMP-enriched fractions from *S. maltophilia* K279a and F60**. Bacterial cultures were performed in TSB and TSB supplemented with 200 μM Dip for 48 h. Outer membrane proteins were extracted as described in the text. Proteins from Sarkosyl-insoluble OMP-enriched fractions (10 μg per lane) were separated by SDS gel electrophoresis according to the Tris/tricine method (Schagger and Von Jagow, 1987) using gradient gels of 4–20%. Gels were stained with Coomassie blue. Lane 1, K279a (TSB); 2, K279a (TSB-Dip); 3, F60 (TSB); 4, F60 (TSB-Dip), and 5: molecular mass markers in kDa. Arrows show two bands, in the range of 70–90 kDa, regulated by iron: Band 1 (higher MW) and Band 2.

With the purpose of detecting *S. maltophilia* iron-repressed outer membrane proteins (IROMP) Sarkosyl-insoluble OMP-enriched fractions from K279a and F60, grown in TSB and TSB-Dip, were compared by SDS-PAGE (Figure 6) Under iron starvation, the SDS-PAGE profile from K279a presented a pattern of two iron-repressed proteins in the range of 70–90 kDa. Band 1 (higher MW) appeared to be stronger under low-iron conditions while Band 2 was only seen when K279a was grown with Dip. The SDS-PAGE profiles from F60, cultured in both conditions, were similar to that of K279a grown with Dip.

Subsequently, Band 1 and Band 2 from K279a were analyzed by mass spectrometry and identified based on the analysis of their mass spectra by Mascot (www.matrixscience.com). Band 1 was matched to the outer membrane receptor FepA from *S. maltophilia* K279a (gi|190573428, YP_001971273, score: 283, protein sequence coverage: 57%). Reference sequence YP_001971273 has been replaced by WP_012479564. The sequence YP_001971273 is 100% identical to WP_012479564 over its full length (749 aa). SMLT_RS06850 (gi|190572091:1472622-1474871) codifies WP_012479564, a protein with a calculated mass of 80.67 KDa.

Band 2 closest match was a colicin I receptor from *S. maltophilia* K279a (gi|190575965, YP_001973810, score: 419, protein sequence coverage: 60%). The old locus tag Smlt4135 (gi|190010013:4245161-4247299) codifies for the putative precursor of colicin I (also known as CirA, YP_001973810), a protein of 712 aa. This locus have been replaced by SMLT_RS19685 (gi|190572091:4245254-4247299) which codifies for the TonB-dependent receptor WP_044570913, a protein of 681 aa with a calculated mass of 73.95 KDa. The sequence YP_001973810 is 100% identical to WP_044570913 over the shared 681 aa.

A BLAST search indicated that the *S. maltophilia* WP_012479564 protein displays closest homology to the outer membrane receptor FepA of *Xanthomonas citri* (Accession: WP_011052402, 66% identity), *P. aeruginosa* (Accession WP_003111874, 50% identity) and *E. coli* (Accession: ADB98042, 47% identity). On the other hand, WP_044570913 showed significant homology to a TonB-dependent receptor of *Pseudomonas putida* (Accession: WP_043199708, 55% identity), a putative colicin I receptor of *Acinetobacter* sp. WC-323 (Accession: EKU56436, 48% identity), a colicin I receptor of *Acinetobacter guillouiae* (Accession: BAP36722, 47% identity), and a colicin IA outer membrane receptor and translocator, ferric iron-catecholate transporter of *E. coli* str. K-12 substr.MG1655 (Accession: NP_416660, 34% identity).

Therefore, by using SDS-PAGE followed by mass spectrometry we identified two IROMPs, WP_012479564, and WP_044570913, which are TonB-dependent receptors. *E. coli* K-12 possesses at least five TonB-dependent receptors for the uptake of different siderophores, including FepA and Cir (also termed CirA). All of them consist of a 22-β-strand barrel formed by ca. 600 C-terminal residues, while ca. 150 N-terminal residues fold inside the barrel to form a hatch, cork or plug domain. The plug domain acts as the channel gate, blocking the pore until the channel is bound by ligand. At this point it undergoes conformational changes which opens the channel (Miethke and Marahiel, 2007). Consequently, we decided to search the protein accession numbers obtained through MASCOT in the conserved domain databases (http://www.ncbi.nlm.nih.gov/Structure/cdd/wrpsb.cgi). The domain hits for WP_012479564 were PRK13524 (multi-domain, Accession PRK13524), outer membrane receptor FepA, for the interval of 28-749 aa (*E*-value 0e+00), and the ligand_gated_channel (Accession cd01347) corresponding to TonB dependent/Ligand-Gated channels in the interval of 52-749 aa (*E*-value 6.06e–122). Furthermore, in the N-terminal plug of ligand_gated_channel (residues 52–171) 54 of 54 of the residues that compose this conserved feature have been mapped to the query sequence. With respect to WP_044570913, the domain hits were FepA (multi-domain, Accession COG4771), outer membrane receptor for ferrienterochelin and colicins, for the interval of 45–681 aa (*E*-value 0e+00), and the ligand_gated_channel (Accession cd01347) in the interval of 66–681aa (*E*-value 2.07e–115). Besides, in the N-terminal plug of ligand_gated_channel (residues 60–172) 54 of 54 of the residues that compose this conserved feature have been mapped to the query sequence. Another hit was PRK13483 (Accession PRK13483) corresponding to enterobactin receptor protein in the interval of 43–681 aa (*E*-value 2.74e–142).

In addition, the SignalP 4.0 program (http://www.cbs.dtu.dk/services/SignalP-4.0) predicts a cleavable N-terminal signal sequence for both *S. maltophilia* proteins, with a potential cleavage site at amino acid 29 for WP_012479564 and at amino acid 35 for WP_044570913.

**Figure 7:**
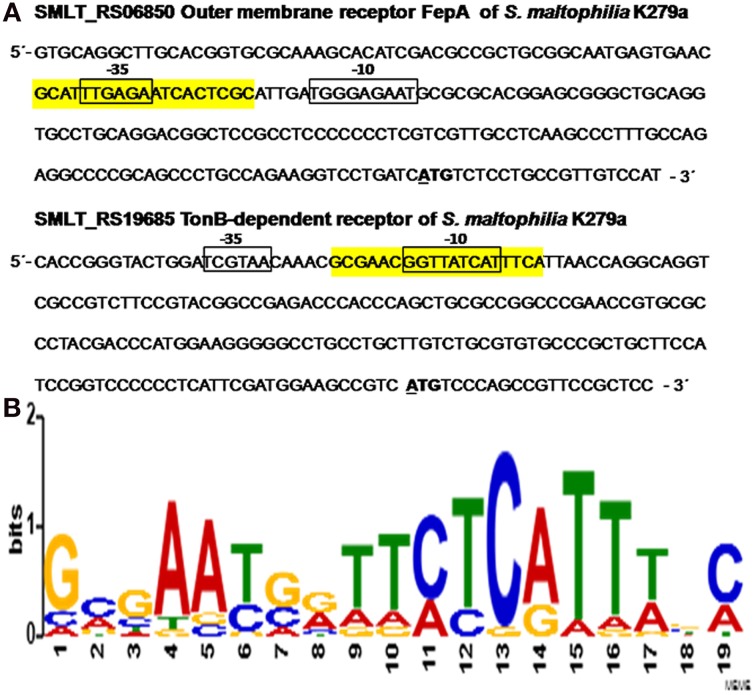
***In silico* analysis of putative Fur boxes of *S. maltophilia* K279a iron regulated genes**. **(A)** Upstream regions (−200 to +21) of SMLT_RS06850 and SMLT_RS19685, coding for the outer membrane receptor FepA and a TonB-dependent receptor, respectively. The -35 and -10 sites of the predicted promoters are shown in boxes and the +1 transcription start sites are in bold and underlined. The putative Fur boxes of SMLT_RS06850 (−144 to −125) and SMLT_RS19685 (−175 to −156) are highlighted in yellow. **(B)** Sequence logo of the putative Fur box of *S. maltophilia*. Detection of potential Fur boxes present in the genome of *S. maltophilia* (GenBank: AM743169.1; (Crossman et al., 2008) was performed with 43 Fur boxes previously characterized (Zaini et al., 2008) using MEME tool. A consensus was built from 20 putative *S. maltophilia* Fur boxes detected with MAST tool.

Since several OMPs which are TonB-dependent receptors are encoded by *fur*-repressed genes (Andrews et al., 2003), the presence of putative Fur-binding sites was searched in the upstream regions of SMLT_RS06850 and SMLT_RS19685, coding for WP_012479564 and WP_044570913, respectively. Firstly, −10 and −35 elements similar to the *E. coli* consensus promoter sequences were identified in the upstream regions (−200 to +21 relative to the start codon) of both genes by using BPROM (Softberry, Inc.) (Figure 7A). Secondly, for the detection of potential Fur boxes in *S. maltophilia* genes a scoring matrix was defined from the 43 Fur binding sites characterized in other bacteria (Zaini et al., 2008) using the MEME tool (http://meme-suite.org). Then, the *S. maltophilia* K279a genome (GenBank: AM743169.1) was analyzed with the resulting scoring matrix using the MAST tool (http://meme-suite.org/tools/mast). Twenty putative *S. maltophilia* Fur boxes (*p* < 10e^−4^) were detected with MAST tool. Figure 7A shows the putative Fur boxes of SMLT_RS06850 (−144 to −125, 5′ GCATTTGAGAATCACTCGC 3′) and SMLT_RS19685 (−175 to −156, 5′ GCGAACGGTTATCATTTCA 3′) identified in a region comprising the −35 element and the −10 element of the respective promoters. In *E. coli*, the iron-bound Fur binds to a well-conserved consensus sequence, known as the Fur box, located in the promoter region of genes directly repressed by Fur (Escolar et al., 1999). Therefore, our results suggest that Fur could regulate the expression of the studied *S. maltophilia* genes in response to iron availability. However, the two identified sequences do not appear to be particularly well conserved since they are identical in 11 over 19 bases to the Fur-box consensus sequence 5′ GATAATGATAATCATTATC 3′ of *E. coli* (de Lorenzo et al., 1987). Escolar et al. (1999) proposed that the intracellular iron concentration and the variability and extension of the sequences targeted by Fur could cause a wide range of responses in each specific case. Some genes undergo mild regulation or coregulation by iron, while others are subjected to a strong repression/induction switch. Figure 7B shows a consensus *S. maltophilia* FUR motif built from 20 putative Fur boxes detected within the 200 bp region upstream a start codon with MAST tool.

With respect to protein functions, in *E. coli* K-12 FepA and CirA are the catecholate siderophore OM receptors for the uptake of Fe-enterobactin and linear Fe-enterobactin degradation products such as dihydroxybenzoyl serine, respectively (Buchanan et al., 2007; Miethke and Marahiel, 2007). We have previously reported the optimization of the chrome azurol S agar assay, based on the addition of Casamino acids and Dip to the CAS medium, for the detection of siderophores in *S. maltophilia*. Moreover, K279a and all local nosocomial isolates studied produced only catechol-type siderophores (Garcia et al., 2012). Interestingly, Ryan et al. (2009) suggested that *S. maltophilia* K279a and R551-3 produce the catechol-type compound enterobactin based on their sequenced genomes. In the genome of *S. maltophilia* K279a, SMLT_RS13395, SMLT_RS13400, SMLT_RS13415, and SMLT_RS13420 encode putative enterobactin synthasa components. According to these data, our results put in evidence the presence of WP_012479564 and WP_044570913, which are TonB-dependent receptors homologs to those related to the uptake of Fe-enterobactin. We are currently performing studies to structurally characterize *S. maltophilia* siderophores.

Little is known about *S. maltophilia* iron uptake systems. Huang and Wong (2007) identified a homolog of the ferric citrate receptor FecA in *S. maltophilia* WR-C, which was induced in the iron-depleted medium supplemented with a low concentration of ferric citrate. Interestingly, their results suggest that this TonB-dependent receptor is regulated by the *rpf* /DSF cell-cell communication system. Consequently, we are going to investigate whether the two iron regulated TonB-dependent receptors described in this study are also regulated by *S. maltophilia* quorum sensing.

### Effect of iron on DSF production

**Figure 8:**
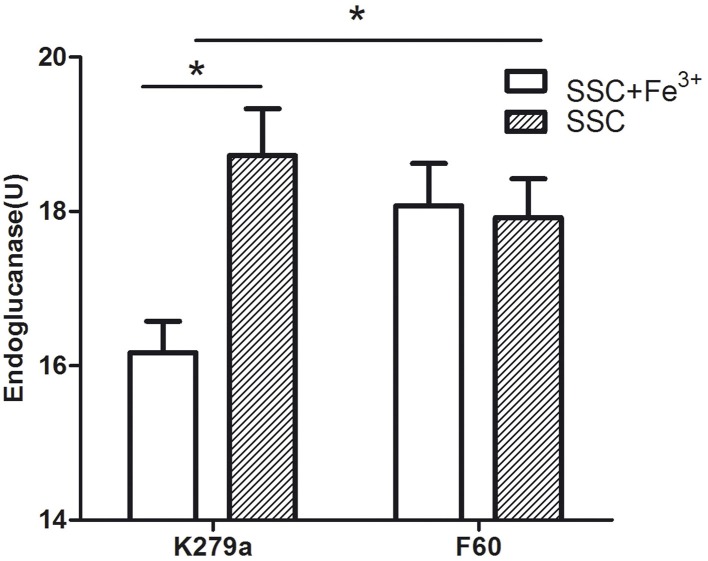
**DSF production by *S. maltophilia* strains grown under iron-limited (SSC) and iron rich conditions (SSC-100 μM FeCl_3_)**. DSF production was assayed by measuring the restoration of endoglucanase activity to the Xcc *rpfF* mutant strain 8523 by culture supernatant extracts obtained using Oasis MAX columns. The results were expressed as units of endoglucanase. Results represent the mean ± standard deviation of one representative experiment. Asterisks indicate significant difference (^*^p < 0.01).

The effect of iron on DSF production was evaluated through the bioassay described by Barber et al. (1997). *S. maltophilia* strains were grown in SSC (iron-limited condition) and in SSC supplemented with FeCl_3_, and DSF present in the supernatants was extracted with Oasis MAX columns. The iron-limited condition cannot be achieved by the addition of Dip, since the chelator present in the extracts inhibits the growth of the reporter strain. Figure 8 shows that the wt strain under the iron-limited condition produced significantly higher amounts of DSF measured as endoglucanase units (18.7 ± 0.6 U) than under the iron rich condition (16.2 ± 0.4 U). On the other hand, the mutant F60 under both conditions, produced higher amounts of DSF (17.9 ± 0.5 and 18.1 ± 0.5 U, respectively) than K279a cultured with 100 μM FeCl_3_. In conclusion, iron, probably through the Fur system, negatively regulates DSF production.

Previous studies, mentioned in the introduction, reported a relationship between Fur and the QS system. In some Gram negative bacteria iron limitation enhances AHL production. This is the first report of the regulation of DSF, an unusual QS signal, by iron, probably through the Fur system.

### Virulence of *S. maltophilia* K279A and F60 in the *Galleria mellonella* infection model

*G. mellonella* has been utilized to study host-pathogen interactions in bacteria, including *Burkholderia cepacia* and *S. maltophilia* (Seed and Dennis, 2008; McCarthy et al., 2011; Nicoletti et al., 2011). *G. mellonella* caterpillars have a humoral immune response which involves melanization and production of antimicrobial peptides, and a cellular response which includes phagocytosis (Hoffmann, 1995). *G. mellonella* is an attractive alternative infection model since its innate immune system shares a high degree of structural and functional homology to that of mammals.

**Figure 9:**
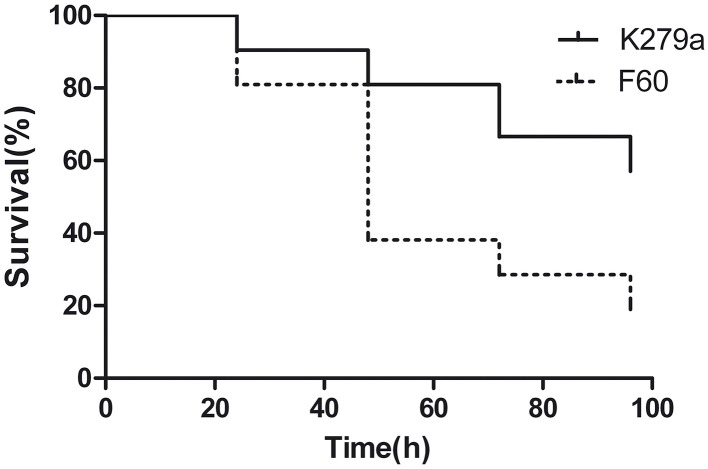
**Virulence of *S. maltophilia* K279a and F60 in the *Galleria mellonella* infection model**. Kaplan-Meier survival curves of *G. mellonella* larvae after 96 h from injection with 1 × 10^5^ UFC/larva of K279a and F60 are shown. Each data set corresponds to a single representative trial with the specified strain (*n* = 12). Differences in survival were calculated using the log-rank test for multiple comparisons and were considered statistically significant at *p* < 0.05. No more than one control larvae injected with sterile physiological saline died in any given trial (not shown).

The influence of the Fur system in *S. maltophilia* virulence was evaluated by infecting larvae of *G. mellonella*. First, we established the optimal inoculum size of *S. maltophilia* required to kill *G. mellonella* over 24 to 96 h by injecting larvae with 10 μl of K279a suspensions containing 10^4^, 10^5^, and 10^6^ CFU. The killing was significantly dependent on the number of *S. maltophilia* cells injected. Inoculation of larvae with 10^4^ CFU of K279a did not produce the killing of any of them after 96 h of infection, whereas the 10^6^ CFU/larva inoculum resulted in the rapid killing of more than 90% of caterpillars within 24 h. On the other hand, the dose of 10^5^ CFU/larva which produced a progressive death of caterpillars over the incubation time was chosen as the optimal inoculum (data not shown). Then, *G. mellonella* killing assays were performed with K279a and F60. Figure 9 shows the corresponding survival curves of a single representative trial. Infection of caterpillars with K279a resulted in 20% of death after 48 h of inoculation, and 43% of death after 96 h, a response significantly different from that obtained with the mutant F60 (*p* < 0.001) which was able to kill 62% of caterpillars after 48 h of inoculation and reached 81% of death after 96 h. No dead caterpillars were detected in the control group. Therefore, the *fur* mutation led to increased virulence to *G. mellonella* larvae, compared to that of its isogenic parental strain, which could be due to the pleitropic effects of this mutation observed in the present study, including increased biofilm formation, and EPS and SOD production.

Very little is known about the pathogenic mechanisms of *S. maltophilia. One* study using the *G. mellonella* infection model suggests that the major extracellular protease StmPr1 may be a relevant virulence factor of *S. maltophilia* (Nicoletti et al., 2011). Another study (McCarthy et al., 2011) using this model reported that Ax21 protein is a cell-cell signal that regulates virulence in *S. maltophilia*. These results and the herein presented show that the *G. mellonella* infection model is a useful tool for future research on *S. maltophilia* virulence.

## Conclusion

The studies described herein are the first to provide evidence about the important role of iron as a signal, likely through the Fur system, for *S. maltophilia* biofilm formation and virulence. For these studies, a spontaneous *fur* mutant was obtained for the first time in *S. maltophilia*. Iron limitation improved biofilm formation and organization, as well as EPS production and SOD activity. Furthermore, MnSOD was responsible for the oxidative stress response of *S. maltophilia*. The *G. mellonella* infection model was useful to evaluate the virulence of the strains used in this work. F60 was more virulent than K279a in the killing assay, in accordance with the described role of iron in the regulation of potential virulence/survival factors. These observations are significant since *S. maltophilia* would be exposed to iron-limiting conditions either in the host or the nosocomial environment.

Moreover, we report the presence of two IROMPs which showed homology with FepA and another putative TonB-dependent siderophore receptor of K279a. *In silico* analysis allowed the detection of potential Fur boxes in the respective coding genes. Additionally, several promoters containing consensus Fur boxes were detected in the genome of K279a, and a consensus *S. maltophilia* FUR motif was built.

This is the first report of the regulation of DSF, an unusual QS signal, by iron, probably through the Fur system. Some authors suggest that iron and QS converge to regulate the expression of some virulence factors while an alternative interpretation could be that QS-regulated traits are likely to be a component of the Fur regulon (Cha et al., 2008). Future additional studies are required to fully define the role of the *S. maltophilia fur* homolog and to characterize the interaction between the QS system and Fur.

Finally, it has been proposed that interference with iron signaling processes could provide an interesting approach to aid the treatment of bacterial infections (Thompson et al., 2012). However, our results as well as those of Wiens et al. (2014) raise concerns about the use of iron chelators in the treatment of CF infections.

## Author contributions

Conceived and designed the study: BP and MF. Performed the experiments and analyzed the data: BP, CG, and EA. Coordinated the study and wrote the manuscript: BP. All authors read and approved the final manuscript.

## Conflict of interest statement

The authors declare that the research was conducted in the absence of any commercial or financial relationships that could be construed as a potential conflict of interest.
